# Effect of *Fusarium*-Derived Metabolites on the Barrier Integrity of Differentiated Intestinal Porcine Epithelial Cells (IPEC-J2)

**DOI:** 10.3390/toxins8110345

**Published:** 2016-11-19

**Authors:** Alexandra Springler, Galina-Jacqueline Vrubel, Elisabeth Mayer, Gerd Schatzmayr, Barbara Novak

**Affiliations:** 1 BIOMIN Research Center, Technopark 1, Tulln an der Donau 3430, Austria; alexandra.springler@biomin.net (A.S.); gala.vrubel@gmail.com (G.-J.V.); e.mayer@biomin.net (E.M.); gerd.schatzmayr@biomin.net (G.S.); 2 Department of Applied Genetics and Cell Biology, University of Natural Resources and Life Sciences, Vienna 1190, Austria

**Keywords:** emerging mycotoxin, transepithelial electrical resistance, IPEC-J2, deoxynivalenol, intestinal barrier function

## Abstract

The human, animal and plant pathogen *Fusarium*, which contaminates agricultural commodities worldwide, produces numerous secondary metabolites. An example is the thoroughly-investigated deoxynivalenol (DON), which severely impairs gastrointestinal barrier integrity. However, to date, the toxicological profile of other *Fusarium*-derived metabolites, such as enniatins, beauvericin, moniliformin, apicidin, aurofusarin, rubrofusarin, equisetin and bikaverin, are poorly characterized. Thus we examined their effects—as metabolites alone and as metabolites in combination with DON—on the intestinal barrier function of differentiated intestinal porcine epithelial cells (IPEC-J2) over 72 h. Transepithelial electrical resistance (TEER) was measured at 24-h intervals, followed by evaluation of cell viability using neutral red (NR) assay. Enniatins A, A1, B and B1, apicidin, aurofusarin and beauvericin significantly reduced TEER. Moniliformin, equisetin, bikaverin and rubrofusarin had no effect on TEER. In the case of apicidin, aurofusarin and beauvericin, TEER reductions were further substantiated by the addition of otherwise no-effect DON concentrations. In all cases, viability was unaffected, confirming that TEER reductions were not due to compromised viability. Considering the prevalence of mycotoxin contamination and the diseases associated with intestinal barrier disruption, consumption of contaminated food or feed may have substantial health implications.

## 1. Introduction 

Mycotoxins are secondary metabolites produced by filamentous fungi, such as those of *Aspergillus*, *Penicillium* and *Fusarium* species [1]. Environmental conditions, improper harvesting, storage and processing operations all contribute to mycotoxin contamination of food and feed, which affects approximately 72% of agricultural commodities worldwide [2]. Mycotoxin occurrence, toxicity and appropriate detoxification strategies have been subject to a great amount of research, although with a primary focus on traditional mycotoxins, including trichothecenes, fumonisins and aflatoxins. The enormous advances of analytical methods and the development of multi-toxin methods have however enabled the detection of a large number of other fungal metabolites. The potential toxicological relevance of these metabolites must therefore be established, and if necessary, appropriate legislative regulations must be put in place [3,4]. The sensitive simultaneous detection of multiple fungal metabolites via liquid chromatography-tandem mass spectroscopy (LC-MS/MS) [5], has aided the discovery of new fungal secondary metabolites, so called “emerging mycotoxins”. These occur frequently and in high concentrations in cereals and cereal-derived products [6,7]. In particular, the *Fusarium* species—a very common agricultural contaminant—produces a large number of secondary metabolites, which occur with varying prevalence. These include compounds such as enniatins, beauvericin, apicidin, aurofusarin and moniliformin [8]. As these have only been discovered over the last few decades, they are—to date—poorly investigated. Consequently, there is a lack of in vitro and in vivo data regarding their toxicological relevance. 

With respect to the latter, the epithelium of the gastrointestinal tract is of particular significance. Due to the oral route of consumption of these compounds through contaminated food and feed, the intestinal epithelium presents the initial barrier of defense and thus a crucial toxicological target. Impairments of the intestinal barrier function lead to increased epithelial permeability and thus the uncontrolled transport of bacterial and viral pathogens, as well as food- and feed-borne toxins. This can trigger nonspecific inflammatory responses and an overstimulation of the gut associated immune system, which can negatively affect parameters such as growth performance and feed conversion rate [9,10]. The harmful effect of mycotoxins on the intestinal epithelial barrier function is particularly well illustrated by the example of the trichothecene deoxynivalenol (DON). Its detrimental effects on epithelial barrier function have been studied in diverse intestinal models, showing decreases in transepithelial electrical resistance (TEER)—an indicator of intestinal barrier integrity—in Caco-2 [11,12], intestinal porcine epithelial cells (IPEC)-1 [13,14] and IPEC-J2 [15,16]. Therefore, the present study—for the first time—identifies potentially harmful effects of other *Fusarium* metabolites on gastrointestinal barrier function. 

Following the request of the European Commission, the European Food Safety Authority (EFSA) has issued a scientific opinion on the risk to human and animal health related to the presence of beauvericin and enniatins in food and feed [17]. According to this document, enniatins were found in 37%, 68% and 76% of food (*n* = 4251), feed (*n* = 3640) and unprocessed grain (*n* = 2647) samples, collected in 12 European countries between 2000 and 2013. In cereal-based products, maximum concentrations were 42, 125, 832 and 980 µg/kg for enniatin A, A1, B, and B1, respectively. In grains, the maximum reported concentrations were 950, 2000, 18,300 and 5720 µg/kg for enniatin A, A1, B, and B1, respectively. Beauvericin was found in 20%, 21% and 54% in food (*n* = 732), feed (*n* = 861) and unprocessed grain (*n* = 554), respectively [17]. Maximum concentrations of beauvericin in grains and in cereal-based food were 6400 and 844 µg/kg, respectively. According to Streit et al. [8], enniatins and beauvericin were found in 96% of feed and 98% of feed raw material samples (both *n* = 83) with median and maximum concentrations of 30 and 5441 μg/kg for enniatins and 6.7 µg/kg and 2330 µg/kg for beauvericin, respectively. Toxicity of enniatins and beauvericin is based on their ionophoric properties, which enables the passage of mono- or divalent cations (e.g., K^+^, Ca^2+^) across cellular membranes, thereby disrupting normal physiological intracellular ion concentrations [18,19,20]. Several in vitro studies have confirmed cytotoxicity of both compounds in the micromolar range [21,22,23,24,25,26]. These report that enniatin- and beauvericin-induced cell death is primarily based on apoptosis induction via the mitochondrial pathway [22,27,28] or necrosis-induction via lysosomal damage [29,30,31]. Contrasting reports have been made regarding enniatin- and beauvericin-induced oxidative stress [32,33,34]. Furthermore, both compounds inhibit the activity of acyl-CoA:cholesterol acyltransferase (ACAT), a protein of the endoplasmic reticulum, which produces cholesteryl esters from cholesterol. The relationship between the ionophoric activity of beauvericin and enniatins and the degree of ACAT inhibition is however still unclear [35]. Studies focusing on in vivo effects of enniatins and beauvericin [36,37,38,39,40,41,42], have confirmed a low degree of toxicity. In the only study to report toxic effects of enniatins, the intraperitoneal (i.p.) administration of 10–40 mg/kg body weight (BW) every 8 h was lethal for immune-deficient mice within 2–5 days. Lower concentrations only caused weight loss [43]. Nevertheless, due to observed contamination levels, further studies are required to investigate chronic toxicity of both compounds, as well as potential combinatory effects due to the high co-occurrence with regulated mycotoxins. In their scientific opinion, the EFSA concluded in 2014 that acute exposure to enniatin and beauvericin is not a concern to human health. However, they further state that due to the lack of relevant in vivo toxicity data, no conclusion can be drawn about the effects of chronic exposure [17].

Similar problems—namely limited toxicity data—also apply to a large amount of other *Fusarium*-derived metabolites, found in cereal and cereal-based products. For example, according to Streit et al. [8], the cyclic histone deacetylate (HDAC) inhibitor apicidin [44,45,46] occured in 66% of analyzed samples (*n* = 83), with median and maximum concentrations of 1.9 µg/kg and 160 µg/kg, respectively. Apicidin alters the acetylation status of histone and non-histone proteins. This leads to chromatin remodeling and epigenetic modification of gene expression, metabolic enzymes and transcription factors [47,48]. Aside from antiproliferative, cyto-differentiation and cytotoxic activity in mammalian cells [44,49,50], apicidin induces toxic effects in rats, including body weight loss, hemorrhage in the stomach, intestines and bladder and finally death [51]. 

Even less is known about aurofusarin and its related orange-brown polyketide rubrofusarin, which occur in 84% and 4% of analyzed samples (*n* = 83), respectively. Median and maximum concentrations were reported to be 85 µg/kg and 17,659 µg/kg for aurofusarin and 2374 µg/kg and 4923 µg/kg for rubrofusarin, respectively. The first characterization of aurofusarin cytotoxicity has only been made recently, showing pronounced effects in Caco-2 cells at 10 µM [52]. With respect to in vivo effects, Dvorska et al. [53,54] reported that the inclusion of aurofusarin in the maternal diet alters antioxidant composition and the fatty acid profile of quail eggs. The same authors reported a deterioration of chicken meat quality through the reduction of fat and protein content [55]. Few studies have investigated the effects of its related metabolite rubrofusarin. These describe rubrofusarin-induced inhibition of human DNA topoisomerase II-α [56] as well as antibiotic effects [40]. Equally little is known about the metabolites equisetin and bikaverin, which occur in 87% and 17% of feed samples (*n* = 83), respectively [8]. Median and maximum concentrations have been reported to reach 23 µg/kg and 13,680 µg/kg for equisetin, and 27,510 µg/kg and 51,130 µg/kg for bikaverin, respectively. Equisetin possesses weak activity against gram-positive bacteria [57], while bikaverin affects different biological processes [58,59,60] by exhibiting inhibitory activity against mammalian cell lines. 

Slightly more is known about the metabolite moniliformin, which, according to Streit et al. [8], occurs in 76% of samples (*n* = 83) of feed and feed raw materials, with median and maximum concentrations of 45 µg/kg and 12,236 µg/kg, respectively. Studies report damaging effects on energy metabolism through impairment of the pyruvate dehydrogenase enzyme complex and subsequent inhibition of the Krebs cycle and cellular respiration [61]. While the in vitro cytotoxic potential of moniliformin depends on the cell line it is applied to [62,63,64], contrasting reports have been made regarding whether [65] or not [66,67] moniliformin possesses genotoxic potential. In vivo studies of moniliformin report far more severe effects than those observed in vitro. Toxic effects have been observed in 1-day-old cockerels (oral) [68], in 9-month-old mink (i.p.) [69], in 7-week female broiler chickens (intravenous (i.v.)) [70], in male and female mice (i.p.) [71], in 7-day-old ducklings (oral) [72] and in female and male rats (oral) [72]. Furthermore, in vivo trials revealed damages to the heart muscle [69,72,73,74], muscular weakness and respiratory distress [72,73], reduced feed intake and weight gain [70,74], as well as impaired immune function [73,75]. 

Despite the natural co-occurrence of food- and feed-borne mycotoxins, the complexity of combination studies frequently leads to the establishment of legal limits, based on single compound studies. For instance, Commission Regulation (EC) 1881/2006 and its amendments regulating maximum levels for certain food contaminants, including fusariotoxins, are based on the assumption that these are present as individual compounds only [76]. However, the above described emerging metabolites are frequently co-produced with well-known fusariotoxins, such as DON [77]. For example, beauvericin and enniatins frequently co-occur with other *Fusarium* derived metabolites in grains and food products. These include culmorin, DON and derivatives, equisetin, fumonisins, fusaproliferin, fusaric acid, fusareon-X, moniliformin, monocerin, nivalenol, T-2/HT-2 toxins and zearalenone and its derivatives [8,78,79,80,81,82,83,84,85,86]. Furthermore, in their analysis of 83 samples of feed and feed raw materials using a multi mycotoxin LC-MS/MS method, Streit et al. [8], reported the co-occurrence of 69 different mycotoxins. According to the authors, mixtures of *Fusarium* toxins are detected frequently. In an analysis of the co-occurrence of regulated, masked and emerging mycotoxins, Kovalsky et al. [6], similarly concluded that the co-occurrence of regulated toxins such as DON was frequent with emerging toxins, such as for example enniatins and moniliformin. According to the EFSA database, DON, DON-3-glucoside, nivalenol, beauvericin and enniatins A, A1, B and B1 were measured in 70 samples of wheat grains of undefined end-use. Enniatin B and B1 were quantified in more than 50% of the data. The prevalence of for enniatin A, A1, beauvericin, nivalenol, DON, and DON-3-glucoside was 1%, 19%, 13%, 4%, 21% and 3%, respectively [17]. Thus, natural co-occurrence of these and other mycotoxins not only implies the need for better knowledge and assessment of their individual toxicology, but highlights the importance of clarifying potential interactions. 

Therefore, we investigated—for the first time—the influence of several fusariotoxins, which frequently co-occur with DON, on the intestinal epithelial integrity of differentiated IPEC-J2. Furthermore, aside from the effects of single fungal metabolites, we examined their influence on intestinal barrier function when combined with an otherwise no-effect concentration of DON. The study delivers valuable information regarding the effect these emerging toxins on a crucial toxicological target, the intestinal epithelium. 

## 2. Results

### 2.1. Deoxynivalenol

We recently demonstrated that DON does not significantly decrease TEER of differentiated IPEC-J2 at concentrations below 5 µM [87]. EC90 values of DON were equal to 8.08 ± 3.15 µM after 24 h, 4.53 ± 0.85 µM after 48 h and 4.45 ± 1.37 µM after 72 h of incubation. The highest concentration used during the present study, i.e., 3 µM, was therefore far below the EC90 of DON after 24, 48 and 72 h.

### 2.2. Enniatins and Combinations of Enniatins

**Figure 1:**
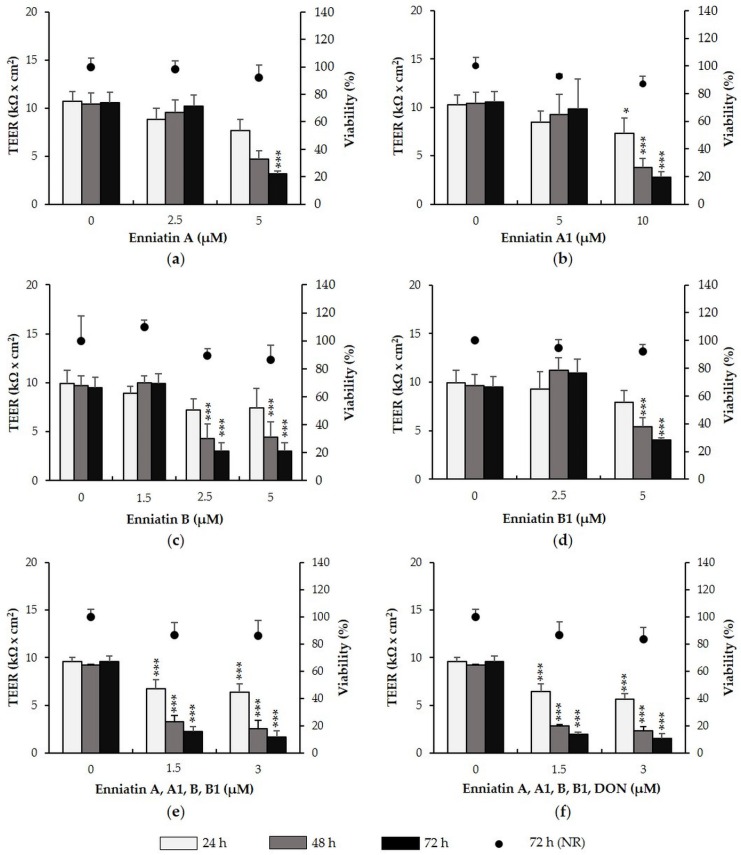
Effect of enniatin A, A1, B and B1 as well as combinations of enniatins (+/− deoxynivalenol, DON) on transepithelial electrical resistance (TEER) and viability of differentiated intestinal porcine epithelial cells (IPEC-J2). IPEC-J2 were treated with (**a**) enniatin A (2.5 and 5 µM); (**b**) A1 (5 and 10 µM); (**c**) B (1.5, 2.5 and 5 µM); or (**d**) B1 (2.5 and 5 µM); as well as (**e**) a combination of all enniatins (each toxin: 1.5 or 3 µM); and (**f**) a combination of all enniatins with DON (each toxin: 1.5 or 3 µM). TEER was measured after 24, 48 and 72 h. After the final TEER measurement, viability was determined via the neutral red (NR) assay. Asterisks indicate significant differences compared to control of the respective time point (* *p* < 0.05, ** *p* < 0.01, *** *p* < 0.001). Data represent mean ± SD, *n* = 3.

Effects of enniatin A, A1, B and B1 on TEER and viability of IPEC-J2 were investigated over 72 h (Figure 1a–d). Exposure of IPEC-J2 to 2.5 µM enniatin A for 72 h had no effect on TEER. At 5 µM, enniatin A had no effect after 24 and 48 h, but significantly reduced TEER by approximately 70% after 72 h (*p* = 0.000). Enniatin A1 did not reduce TEER at 5 µM, showing effects only at 10 µM after 24 h (−29%, *p* = 0.032), 48 h (−64%, *p* = 0.003) and 72 h (−74%, *p* = 0.009). Treatment of IPEC-J2 with enniatin B (1.5, 2.5, and 5 µM) did not affect TEER after 24 h, but significantly reduced TEER at 2.5 and 5 µM after 48 h (both −55%, *p* = 0.002) and 72 h (both −68%, *p* = 0.000). Enniatin B1 did not reduce TEER at 2.5 µM over 72 h, however induced significant decreases at 5 µM after 48 h (−44%, *p* = 0.007) and 72 h (−58%, *p* = 0.001). Enniatin B had the strongest effect on TEER, followed by enniatin B1, A and A1.

We next assessed the effect of a combination of enniatins A, A1, B and B1 on TEER of IPEC-J2 in the absence (Figure 1e) or presence of DON (Figure 1f). A combination of all enniatins, containing 1.5 µM or 3 µM of each individual toxin, led to significant TEER decreases after 24 h (1.5 µM: −30%, *p* = 0.008; 3 µM: −33%, *p* = 0.000), 48 h (1.5 µM: −64%, *p* = 0.000; 3 µM: 72%, *p* = 0.000) and 72 h (1.5 µM: −75%, *p* = 0.000; 3 µM: −82%, *p* = 0.000). The combination of enniatins, which if applied individually show no effect on TEER, seem to have an additive effect if applied as a combination. Addition of DON to the enniatin combinations did not further substantiate enniatin-induced TEER decreases. The combination of all enniatins and DON, with each toxin present at 1.5 or 3 µM, significantly reduced TEER after 24 h (1.5 µM: −33%, *p* = 0.001; 3 µM: −41%, *p* = 0.000 ), 48 h (1.5 µM: −69%, *p* = 0.000; 3 µM: −75%, *p* = 0.003), and 72 h (1.5 µM: −80%, *p* = 0.000; 3 µM: −83%, *p* = 0.000). 

To exclude that TEER reductions were due to cytotoxic effects, a neutral red (NR) assay was conducted after the final TEER measurement (72 h) (Figure 1a–f). Viability of IPEC-J2 treated with individual enniatins or with combinations of enniatins (+/− DON) was unaffected at all test concentrations. 

### 2.3. Beauvericin

**Figure 2:**
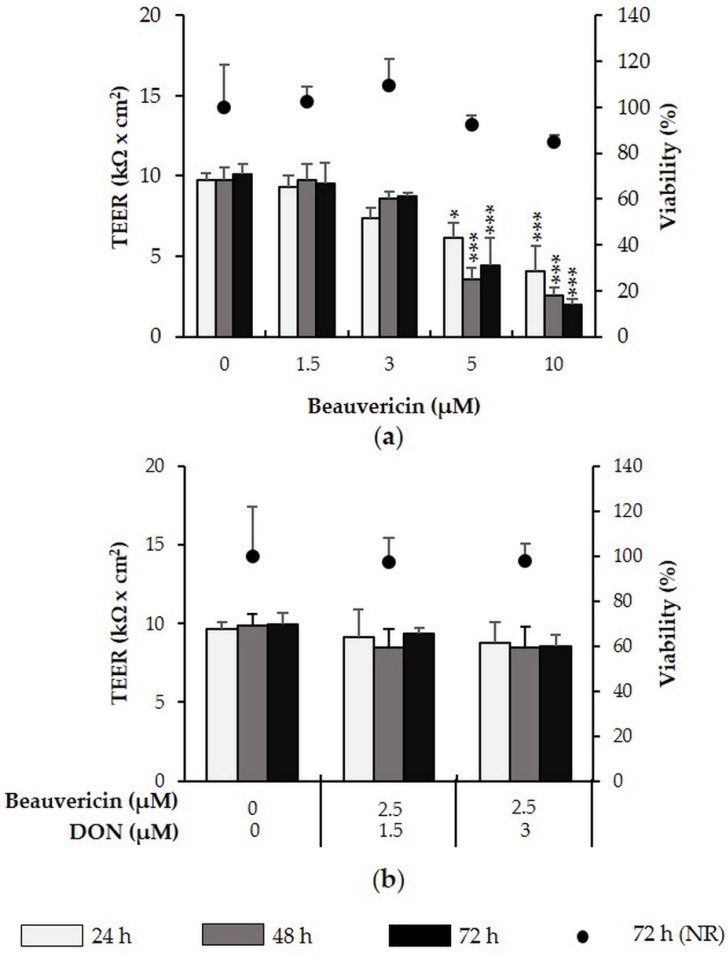
Effect of beauvericin (+/− DON) on TEER and viability of differentiated intestinal porcine epithelial cells (IPEC)-J2. IPEC-J2 were treated with (**a**) beauvericin (1.5–10 µM) as well as (**b**) a combination of beauvericin (2.5 µM) and DON (1.5 or 3 µM). TEER was measured after 24, 48 and 72 h. After the final TEER measurement, viability was determined via the NR assay. Asterisks indicate significant differences compared to control of the respective time point (* *p* < 0.05, ** *p* < 0.01, *** *p* < 0.001). Data represent mean ± SD, *n* = 3.

We next assessed the effect of beauvericin (1.5–10 µM) on barrier integrity of IPEC-J2 (Figure 2a). While 1.5 and 3 µM beauvericin had no effect on IPEC-J2, 5 and 10 µM significantly reduced TEER after 24, 48 and 72 h. Maximum TEER reductions were observed at 10 µM beauvericin, with reductions of 59% after 24 h (*p* = 0.000), 74% after 48 h (*p* = 0.000) and 80% after 72 h (*p* = 0.000). Combination of a no-effect beauvericin concentration (2.5 µM) with 1.5 µM or 3 µM DON, did not lead to a significant reduction of TEER (Figure 2b). In all cases, viability was not significantly reduced at any concentration. 

### 2.4. Apicidin

**Figure 3:**
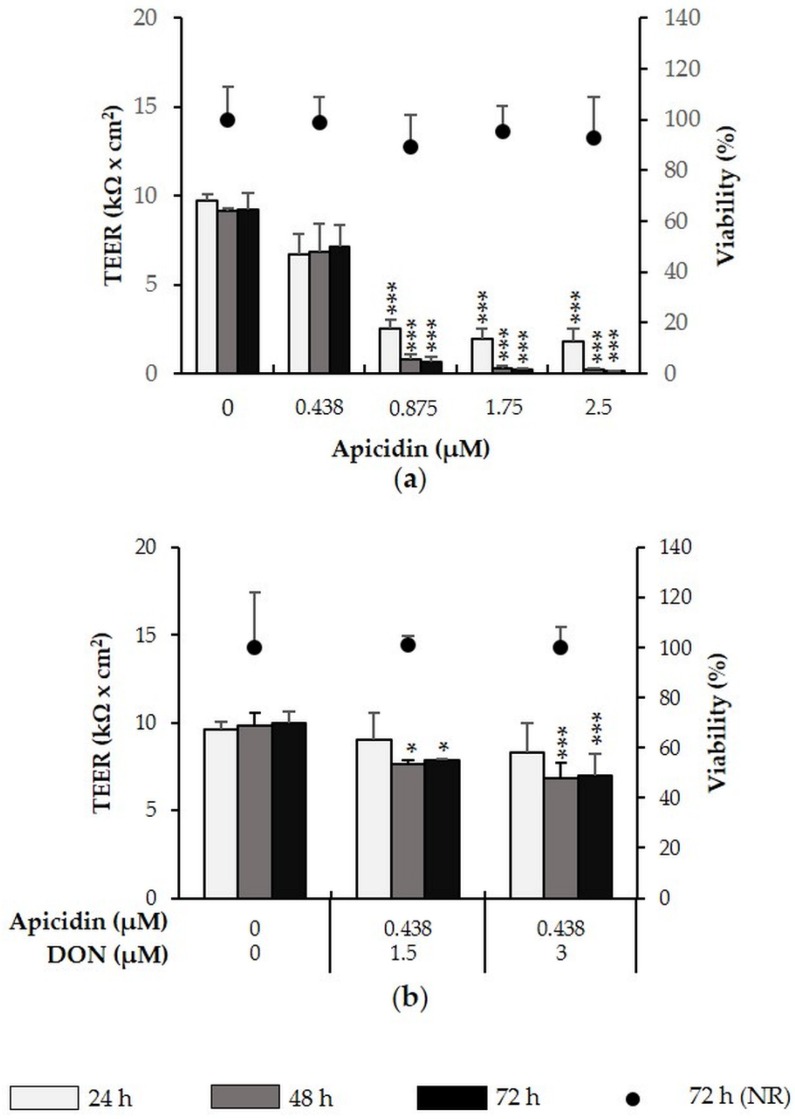
Effect of apicidin (+/− DON) on TEER and viability of differentiated IPEC-J2. Differentiated IPEC-J2 were treated with (**a**) apicidin (0.438–2.5 µM) or (**b**) a combination of apicidin (0.438 µM) and DON (1.5 or 3 µM). TEER was measured after 24, 48 and 72 h. After the final TEER measurement, viability was determined via the NR assay. Asterisks indicate significant differences compared to control of the respective time point (* *p* < 0.05, ** *p* < 0.01, *** *p* < 0.001). Data represent mean ± SD, *n* = 3.

Examination of the effect of apicidin (0.438–2.5 µM) (Figure 3a), showed that while 0.438 µM left TEER unaffected, 0.875–2.5 induced a time- and dose-dependent TEER decrease. At the maximum tested concentration of 2.5 µM apicidin, TEER was decreased by 93% (*p* = 0.006) (24 h), 98% (*p* = 0.000) (48 h) and 99% (*p* = 0.008) (72 h). The combination of a no-effect apicidin concentration (0.438 µM) and DON (1.5 or 3 µM) significantly reduced TEER after 48 and 72 h, showing an additive effect. The combination with 1.5 µM DON reduced TEER by approximately 23% (*p* = 0.013) after 48 h and 21% (*p* = 0.003) after 72 h. The combination of 0.438 µM apicidin with 3 µM DON reduced TEER by 31% after 48 h (*p* = 0.032) and 27% (*p* = 0.009) after 72 h (Figure 3b).

Neither apicidin alone nor the combination of apicidin with DON reduced IPEC-J2 viability after 72 h.

### 2.5. Aurofusarin

**Figure 4:**
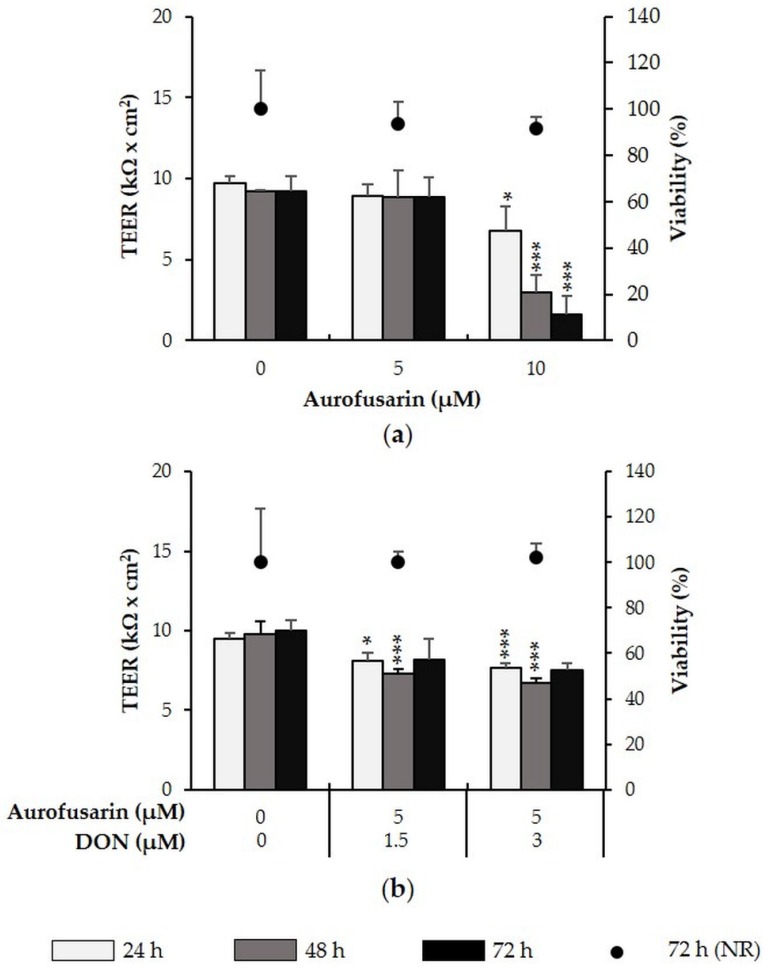
Effect of aurofusarin (+/− DON) on TEER and viability of differentiated IPEC-J2. IPEC-J2 were treated with (**a**) aurofusarin (5–10 µM) as well as (**b**) a combination of aurofusarin (5 µM) with DON (1.5 or 3 µM). TEER was measured after 24, 48 and 72 h. After the final TEER measurement, viability was determined via the NR assay. Asterisks indicate significant differences compared to control of the respective time point (* *p* < 0.05, ** *p* < 0.01, *** *p* < 0.001). Data represent mean ± SD, *n* = 3.

Exposure to 5 µM aurofusarin did not affect TEER of IPEC-J2 over 72 h. At 10 µM, aurofusarin significantly reduced TEER after 24 h (−30%, *p* = 0.019), 48 h (−68%, *p* = 0.001) and 72 h (−83%, *p* = 0.000) (Figure 4a). When combined with DON (1.5 or 3 µM), TEER of IPEC-J2 was significantly decreased after 24 and 48 h. The combination of an otherwise no-effect aurofusarin concentration (5 µM) with DON (1.5 µM) significantly reduced TEER by 15% (*p* = 0.013) after 24 h and by 25% (*p* = 0.002) after 48 h. Aurofusarin (5 µM) combined with 3 µM DON reduced TEER by 20% after 24 h (*p* = 0.000) and by 32% (*p* = 0.001) after 48 h (Figure 4b). The combination of aurofusarin and DON therefore seems to have an additive effect. No significant effects were observed after 72 h. In all cases, viability of IPEC-J2 remained unaffected.

### 2.6. Metabolites with No Effect on TEER

**Figure 5:**
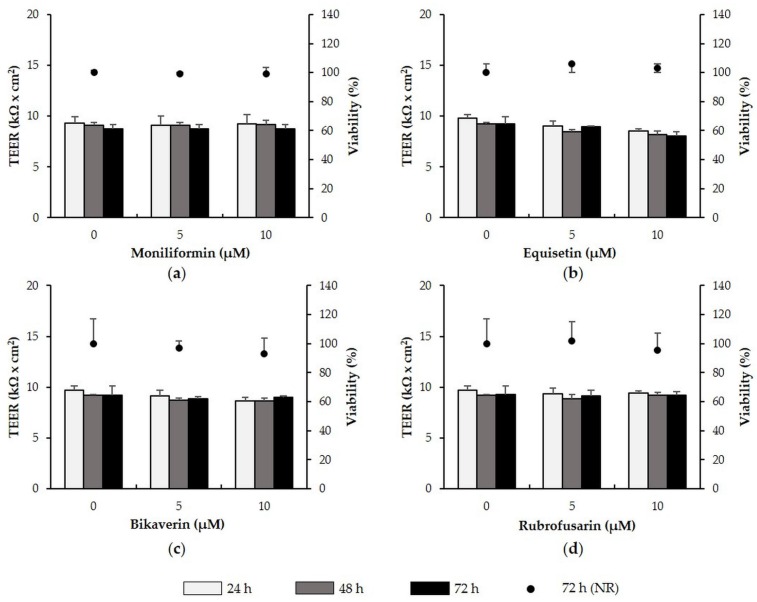
Effect of moniliformin, equisetin, bikaverin and rubrofusarin on TEER and viability of differentiated IPEC-J2. IPEC-J2 were treated with (**a**) moniliformin (5–10 µM); (**b**) equisetin (5–10 µM); (**c**) bikaverin (5–10 µM); and (**d**) rubrofusarin (5–10 µM). TEER was measured after 24, 48 and 72 h. Following the final TEER measurement, viability was determined via the NR assay. Data represent mean ± SD, *n* = 3.

Exposure of IPEC-J2 to moniliformin, equisetin, bikaverin and rubrofusarin (all: 5 and 10 µM) did not affect TEER or viability over a time period of 72 h (Figure 5a–d).

## 3. Discussion

Advanced analytical methods have enabled the discovery of numerous new *Fusarium*-metabolites, most of which have received little to no investigatory attention. In particular, their effect on the intestinal barrier function has so far been neglected. Thus, we herein present the first investigation of the effect of such metabolites on intestinal barrier integrity of intestinal porcine epithelial cells. Such effects are well documented for the trichothecene mycotoxin DON [11,15,87,88,89], as well as for aflatoxins [90,91], zearalenone [92,93], ochratoxin A [94,95], patulin [96,97], fumonisin B1 [98,99,100] and T2 toxin [16,101]. Similarities with the original in vivo cell tissue and with humans and the fact that pigs are the most susceptible species to *Fusarium* mycotoxin DON, make porcine cell lines a highly suitable option for in vitro analysis of intestinal barrier integrity [102]. IPEC-J2 [103], possess strong morphological and functional resemblance of intestinal epithelial cells in vivo and have recently been reported morphologically and functionally even more differentiated than its related cell line IPEC-1 [104].

With respect to enniatins, the use of the IPEC-J2 cell line, isolated from the jejunum of neonatal piglets, is of particular significance. In an analysis of the distribution of enniatins in different rat tissues, Rodriguez-Carasco et al. [36] and Manyes et al. [38] reported the highest levels of the compounds in the jejunum, liver and fat tissue, concluding that the jejunum is a significant site of enniatin absorption. The enniatins investigated in this study were chosen due to their natural occurrence in food and feed. In numerous European countries, contamination levels range from µg/kg to mg/kg in cereal grains and unprocessed food. According to Malachova et al. [82], 73% of cereal-based products in the Czech Republic were contaminated with enniatins A, A1, B and B1. In the remaining samples, at least three of the four mycotoxins were identified. Furthermore, 89% of Finish grain samples were co-contaminated with enniatins A, A1, B and B1, of which enniatin B and B1 occurred most frequently in commercial samples [80]. In Spain, up to 82% of available cereal products are co-contaminated by enniatins [105]. Enniatins are not legislated—no estimated daily intake (TDI) or provisional maximum tolerable daily intake (PMTDI) has been proposed by authorities. The choice of realistic in vitro concentrations is therefore difficult. Concentrations of enniatins used for this study were based on an approach by Prosperini et al. [106], who concluded that for other fusariotoxins such as fumonisins, the maximum levels in raw cereals reach approximately 2000 µg/kg. Taking this value into consideration and considering cereal consumption in the European Union to be 125.4 kg/year, the group estimated that Europeans consumed approximately 680 µg fumonisins per day, and applied this calculation to enniatins, for example enniatin A1. Following this approach, ingestion levels were estimated to approximately equal 1.01 µM in vitro. In addition, it is known that enniatins are particularly stable in the gastrointestinal tract [27], suggesting accumulation of the metabolites. Considering this and the fact that enniatin concentrations in food additionally depend on dietary food variety, we applied enniatin concentrations ranging up to 10 µM. 

While all tested enniatins reduced TEER of differentiated IPEC-J2 in the micromolar range, sensitivities to the toxins varied. IPEC-J2 were most sensitive to enniatin B, followed by enniatin B1, A and A1. To date, no other reports exist regarding the effect of enniatins on TEER. However, the negative effect, as observed in this study, could inherently be related to the typical ionophoric properties of enniatins, which facilitate the transport of mono- or divalent cations such as K^+^ or Ca^2+^ across membranes. According to Tai et al. [107], an increase in intracellular Ca^2+^ through the ionophore A23187, decreased tight junction (TJ) resistance in T84 monolayers. Authors speculate that this intracellular Ca^2+^ effect was mediated by protein kinase C. Similarly, Kan et al. [108], demonstrated that increases of intracellular Ca^2+^ via the ionophore A23187, significantly increases TJ permeability in rat liver. 

A comparable explanation could apply to beauvericin, which reduced TEER of differentiated IPEC-J2 at 5 and 10 µM after 24, 48 and 72 h. The choice of a suitable beauvericin concentration was based on previous in vitro studies, which report that, like enniatins, beauvericin elicits its toxicity via ionophoric properties in the low micromolar range [21,22,23,25]. It has recently been reported, that beauvericin (3 and 10 µM) induces expression of phosphorylated mitogen activated protein kinase (MAPK) ERK44/42 within 24 h of exposure [109]. This is a documented feature of DON, where impairments of barrier function—shown by TEER decreases and compromised expression of TJ proteins claudin-3 and 4—are mediated through p44/42 phosphorylation [13,87]. It seems plausible that a potential beauvericin-induced activation of p44/42 could at least partially be responsible for the observed reductions of TEER in beauvericin-treated IPEC-J2.

Negative effects on TEER were also observed by the metabolites apicidin and aurofusarin. Few in vitro cell-based studies are dedicated to these metabolites. Han et al. [110] for example, tested the effect of apicidin on cell proliferation of diverse cell lines via the sulforhodamine B assay and reported IC50 concentrations ranging between approximately 160 nM and 3.8 µM. Furthermore, Bauden et al., [44] reported cytotoxicity of apicidin in pancreatic cancer cells at 100 nM after 48 h. Consequently, apicidin was applied to differentiated IPEC-J2 at low concentrations ranging between 0.438 to 2.5 µM. Profound TEER-reducing effects of apicidin were already observed at 0.875 µM after 24, 48 and 72 h. Apicidin-induced TEER reductions are particularly interesting with respect to its ability to inhibit HDAC and recent reports made by Krishnan et al. [111]. Authors of this study reported that sodium butyrate and trichostatin A, two structurally different HDAC inhibitors, significantly inhibited the expression of TJ protein claudin-1 in colon cancer cells, seemingly through the modulation of claudin-1 mRNA stability. Although no studies have investigated the effect of apicidin on TJ expression, its properties as a HDAC inhibitor could contribute to its negative effect on TEER, an inherent measure of TJ intactness. Analysis of aurofusarin and its related orange-brown polyketide rubrofusarin on TEER of IPEC-J2 revealed negative effects of aurofusarin, not however of rubrofusarin. Little is known regarding the toxicology of these compounds. Based on a recent study of aurofusarin, which revealed strong cytotoxic effects at 10 µM in Caco-2 cells [52], both metabolites were applied to cells up to a concentration of 10 µM. In accordance with the latter study, we first report aurofusarin-induced TEER reductions at 10 µM at all three time points. Interestingly, Dvosrka et al. [53,54,112], reported that the inclusion of aurofusarin in the maternal diet induces changes in antioxidant composition and fatty acid profile of quail eggs. Thus, one could speculate that possibly the lipid bilayer of the cell membrane, in particular its phospholipids, could pose as a toxicological target of aurofusarin. Membrane defects could in turn negatively affect measured TEER values. 

We furthermore report that the metabolites moniliformin, equisetin and bikaverin do not negatively affect TEER of differentiated IPEC-J2. The lack of a negative in vitro effect of moniliformin is in accordance with observations reported in literature. Although numerous in vivo studies have revealed severe effects of moniliformin in diverse species [68,70,75], in vitro effects have been described as mild [62,113]. It has been suggested that the incomplete uptake of the polar toxin through the cell membrane in cell cultures studies may be a possible explanation for this discrepancy. 

Although the investigation of their individual effects is significant and an important prerequisite, the natural co-contamination of *Fusarium*-derived metabolites in food and feed [2,8], additionally calls for the evaluation of combined effects. In fact, mycotoxins of similar structure or those produced by the same species or families, are likely to exhibit a similar mode of action and may therefore exert additive effects [114]. Enniatins, for example, most frequently occur in combinations. We therefore treated IPEC-J2 with a mixture of enniatins A, A1, B and B1, with each toxin being present at 1.5 µM and 3 µM, i.e., no-effect concentration if applied individually. These combinations led to highly significant reductions of TEER after 24, 48 and 72 h, pointing towards an additive effect. This is in accordance with Prosperini et al. [106], who tested a quaternary mixture of enniatin A, A1, B and B1 on viability of Caco-2 cells, with each toxin applied at 0.3125, 0.75, 1.25, or 2.5 µM. Authors concluded a general additive effect.

Furthermore, enniatins as well as other *Fusarium*-derived metabolites frequently co-occur with other primary mycotoxins, such as DON [6]. We recently reported that DON concentrations below 5 µM do not negatively affect TEER of differentiated IPEC-J2 over a duration of 72 h [87]. Therefore we combined no-effect concentrations of metabolites (if applied individually) with no-effect DON concentrations (1.5 and 3 µM). With regard to enniatins, this did not additionally increase the effect of the enniatin mixture on TEER of IPEC-J2. Similarly, the combination of a no-effect beauvericin concentration with a no-effect DON concentration, left TEER of differentiated IPEC-J2 unaffected. Only one study, focusing on DNA fragmentation in Caco-2 cells, reported additivity of beauvericin and DON. In the same study authors additionally reported synergistic effects with regard to lipid peroxidation [115]. Ficheux et al. [62] observed synergism between beauvericin and DON in terms of cytotoxicity in human hematopoietic progenitors. Finally, Ruiz et al. [116,117] identified antagonistic cytotoxic effects of beauvericin and DON in Chinese hamster ovary and monkey kidney epithelial cells. With respect to apicidin and aurofusarin, we found that the combination of a no-effect apicidin concentration with DON, as well as a no-effect aurofusarin concentration with DON, led to significant TEER reductions. This hints at an additive interaction and raises the significance of these toxins due to their natural co-occurrence. In this context, we certainly acknowledge the limitation of our study with regard to being able to draw firm conclusions about combinatory effects. We are aware that to able to firmly conclude additive, synergistic or antagonistic effects, it is necessary to conduct an analysis according to the isobologram method of Chou [118] and Chou and Talalay [119]. Nevertheless, we provide a first insight into potential comminatory effects of these metabolites, especially with regard to their interaction with DON and highlight the need for further investigation into this direction.

Despite the effects observed on TEER, viability of IPEC-J2 remained unaffected throughout all experiments. None of the tested metabolites, whether they reduced TEER or not, negatively affected IPEC-J2 viability. Thus, it can be concluded, that TEER reductions were not a result of cell death. Although, for all tested metabolites of this study, we are the first to report viability data in differentiated IPEC-J2, cytotoxicity of several metabolites has been studied in alternative cell models. With respect to enniatins and beauvericin, studies repeatedly report cytotoxicity at micromolar concentrations in diverse cell types [21,22,23,25,77]. Only few reports have been made regarding in vitro cytotoxicity of apicidin, with strong deviations depending on the cells used [49]. In accordance with our findings, in vitro investigations of moniliformin report a low degree of cytotoxicity [113,120]. Furthermore, only one study has shown cytotoxicity of 10 µM aurofusarin in Caco-2 cells [53], which is in agreement with our finding. With regard to equisetin, rubrofusarin and bikaverin, no cytotoxicity reports are available. 

## 4. Conclusions

Therefore, we present the very first analysis of the influence of several *Fusarium*-derived metabolites, which frequently co-occur with DON, on the intestinal epithelial barrier integrity of differentiated IPEC-J2. We demonstrated that while enniatins A, A1, B and B1, as well as beauvericin, apicidin, and aurofusarin severely reduced intestinal barrier integrity of differentiated IPEC-J2, other metabolites such as moniliformin, equisetin, rubrofusarin and bikaverin had no effect (apicidin > enniatin B > beauvericin = DON > enniatin B1 > enniatin A >aurofusarin = enniatin A1 > moniliformin = equisetin = bikaverin = rubrofusarin). We additionally provide—for the first time—cytotoxicity data of these metabolites in the intestinal porcine epithelial cells. Furthermore, aside from the effects of single mycotoxins, we provide insights into potential additive effects of enniatins, beauvericin, apicidin and aurofusarin with DON. The study substantially adds to the current knowledge of the toxicology of these so-called emerging toxins with respect to a crucial toxicological target, the intestinal epithelium. It presents an interesting starting point for further evaluations, for instance regarding effects of these metabolites on tight junctions or MAPK activity. In preliminary trials we were able to show that the selected metabolites—just like DON [87]—do not reduce protein expression of zona occludens (ZO)-1 and occludin. However, preliminary work suggests effects on the expression of tight junction protein, claudin-1 (data not shown). A similar effect was seen for DON in Springler et al. [87]. Further investigations into this direction are required. The collection of in vivo data of these compounds in pigs, especially in combination with the most common mycotoxin DON, presents an urgent need. Particularly their effect on intestinal structure and function is of great interest, since the so called “leaky gut” can be associated with a particularly high susceptibly to diseases as well as reduced growth performance. 

## 5. Materials and Methods

### 5.1. Cell Culture

The non-transformed intestinal porcine epithelial cells IPEC-J2 (ACC 701; Leibnitz Institute DSMZ, German Collection of Microorganisms and Cell Cultures, Braunschweig, Germany) were continuously maintained in complete cultivation medium consisting of Dulbecco’s modified eagle medium (DMEM/Ham’s F12 (1:1), Biochrom AG, Berlin, Germany), supplemented with 5% fetal bovine serum, 1% insulin-transferrin-selenium, 5 ng/mL epidermal growth factor, 2-5 mM Glutamax (all Gibco^TM^, Life Technologies, Carlsbad, CA, USA) and 16 mM 4-(2-hydroxyethyl)-1-piperazineethanesulfonic acid (Sigma-Aldrich, St. Louis, MO, USA) at 39 °C and 5% CO_2_. Mycoplasma tests were performed regularly via PCR to confirm that cells were free of mycoplasma contamination (Venor^®^ GeM Mycoplasma Detection Kit; Minvera Biolabs GmbH, Berlin, Germany). Cells were routinely seeded at 1 × 10^6^ cells/mL in 150 cm^2^ tissue culture flasks (Eppendorf, Hamburg, Germany) with 28 mL complete cultivation medium and subcultured upon confluence every 4 days for a maximum of 15 passages. For assays, confluent cells were detached using Trypsin (0.25%)-ethylenediaminetetraacetic acid (EDTA, 0.5 mM).

### 5.2. Chemicals

Deoxynivalenol (DON, from *Fusarium* sp., ≥95%, Biopure, RomerLabs^®^, Tulln, Austria), was dissolved in sterile distilled water to produce a stock concentration of 6.75 mM and was further diluted to the desired working concentrations in complete cultivation medium. Enniatin A, A1, B and B1 (all from *Gnomonia* errabunda, ≥95%), as well as moniliformin (from *Fusarium* proliferatum, ≥98%), all from Sigma-Aldrich, St. Louis, MO, USA, and aurofusarin (from *Fusarium* graminearum, ≥97%), equisetin (from *Fusarium* equiseti, ≥99%) and bikaverin (from *Fusarium* sp., ≥95%), all from Santa Cruz Biotechnology Inc., Dallas, TX, USA, were prepared in dimethyl sulfoxide (DMSO) as a stock solution of 1 mg/mL. Beauvericin (from *Beauveria* sp., ≥97%) and rubrofusarin (from *Fusarium* graminearum, ≥98%), both from Adipogen International, Liestal, Switzerland, as well as apicidin (from *Fusarium* sp., ≥95%, Santa Cruz Biotechnology Inc., Dallas, TX, USA) were prepared in DMSO as stock solution of 5 mg/mL. Dilutions to the desired working concentrations were made in complete cultivation medium. DMSO concentrations ins sample dilutions did not exceed a concentration of 1%. Effects of this DMSO concentration on experimental parameters were excluded (Figure S1).

### 5.3. Measurement of Transepithelial Electrical Resistance (TEER)

IPEC-J2 were seeded in the apical compartment of 1.12 cm^2^ Transwell^®^ polyester membrane inserts with 0.4 µM pores (Corning Inc., New York, NY, USA) at a seeding density of 1 × 10^5^ cells/insert and differentiated for 8 days, as previously described by our group. Subsequently, cells were treated with either enniatin A (2.5 and 5 µM), A1 (5 and 10 µM), B (1.5, 2.5 and 5 µM), B1 (2.5 and 5 µM), beauvericin (1.5–10 µM), apicidin (0.438–2.5 µM), aurofusarin, moniliformin, equisetin, bikaverin or rubrofusarin (all: 5 and 10 µM). Concentrations were based on available literature reports. Alternatively, metabolite combinations were tested, with each metabolite being present at a no-effect concentration if applied individually. Cells were treated with a combination of enniatins A, A1, B and B1 (each toxin: 1.5 or 3 µM), a combination of enniatins A, A1, B, B1 and DON (each toxin: 1.5 or 3 µM), a combination of apicidin (0.438 µM) and DON (1.5 or 3 µM), a combination of aurofusarin (5 µM) and DON (1.5 or 3 µM), and a combination of BEA (2.5 µM) and DON (1.5 or 3 µM). Untreated cells served as negative control in each experiment. TEER was measured after 24, 48 and 72 h, using a Millicell-Electrical Resistance System (ERS, Merck Millipore, Billerica, MA, USA). The unit area resistance was obtained by multiplying the meter reading (Ohm) by the surface area of the Transwell^®^ membrane (1.12 cm^2^). The resulting dimension was Ohm × cm^2^. 

### 5.4. Viability Assay

After the final TEER measurement, viability of IPEC-J2 was examined via the neutral red (NR) assay (Aniara, West Chester, OH, USA). At the end of the 72-h incubation period, treatments were removed and cells were washed with a washing buffer provided by the assay kit. According to the manufacturer’s instructions, NR was added to the negative control and test inserts at a final dilution of 1:100 in complete cultivation medium at 39 °C for 3 h. Afterwards cells were fixed for 1 min with the provided fixing solution and incubated with the provided solubilization solution at room temperature for 15 min. Next, 100 µL of each insert was transferred to individual wells of a fresh 96-well plate (Eppendorf, Hamburg, Germany). The quantity of dye incorporated into cells was measured photometrically at 540 nm, with a reference wavelength of 690 nm, using a microplate reader (Biotek, Winoosky, VT, USA). The obtained optical density (OD) is directly proportional to the number of viable cells. Results were calculated as percent viability normalized to the negative control, which was set to 100%.

### 5.5. Statistical Analysis

Statistical analysis was performed with IBM^®^ SPSS Statistics (Version 19.0, IBM corp., New York, NY, USA, 2010). Values of each independent experiment were expressed as means of triplicates ± standard deviation (SD) of three independent experiments. All values were analyzed for normality (Shapiro-Wilk) as well as homogeneity of variance (Levene Statistics). Normally distributed homogenous data were analyzed by analysis of variance (ANOVA) and subsequently via the Dunnett’s *t*-test. If data were normally distributed but not homogenous, ANOVA and Dunnett’s T3-test was used. If normal distribution was violated, the Kruskall-Wallis Test was used. 

## Supplementary Materials

The following are available online at www.mdpi.com/2072-6651/8/11/345/s1, Figure S1: Effect of DMSO (1%) on TEER and viability of differentiated IPEC-J2. Differentiated IPEC-J2 were treated with DMSO (1%). TEER was measured after 24, 48 and 72 h. After the final TEER measurement, viability was determined via the NR assay. Data represent mean ± SD, *n* = 3.

## Abbreviations

The following abbreviations are used in this manuscript:

ACAT	acyl-CoA:cholesterol acyltransferase
BW	body weight
DMEM	Dulbecco’s modified Eagle’s medium
DMSO	dimethyl sulfoxide
DON	deoxynivalenol
EFSA	European food safety authority
HDAC	histone deacetylase
i.p.	intraperitoneal
i.v.	intravenous
IPEC	intestinal porcine epithelial cells
LC-MS	liquid chromatography-mass spectrometry
MAPK	mitogen activated protein kinase
NR	neutral red
OD	optical density
TEER	transepithelial electrical resistance
TJ	tight junction
