# Supplementary material for: Effect of Fusarium-Derived Metabolites on the Barrier Integrity of Differentiated Intestinal Porcine Epithelial Cells (IPEC-J2)
Source: Toxins (Basel). 2016 Nov 19;8(11):345. doi: 10.3390/toxins8110345 (PMC5127141; doi:10.3390/toxins8110345)
Supplement: Supplementary file 1 [file toxins-08-00345-s001.pdf]

Supplementary Materials: Effect of *Fusarium*-Derived Metabolites on the Barrier Integrity of Differentiated Intestinal Porcine Epithelial Cells (IPEC-J2)

Alexandra Springler, Galina-Jacqueline Vrubel, Elisabeth Mayer, Gerd Schatzmayr and Barbara Novak

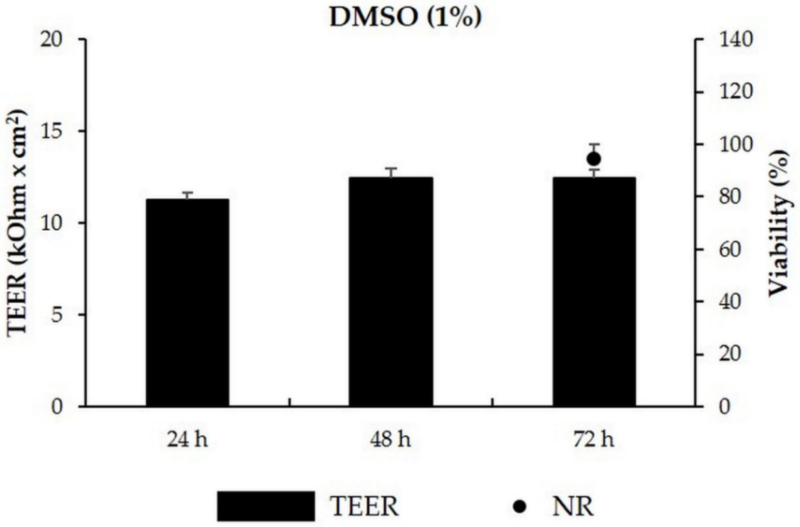

Figure S1. Effect of DMSO (1%) on TEER and viability of differentiated IPEC-J2. Differentiated IPEC-J2 were treated with DMSO (1%). TEER was measured after 24, 48 and 72 h. After the final TEER measurement, viability was determined via the NR assay. Data represent mean ± SD, *n* = 3.
